# Splenic Dendritic Cells and Macrophages Drive B Cells to Adopt a Plasmablast Cell Fate

**DOI:** 10.3389/fimmu.2022.825207

**Published:** 2022-04-12

**Authors:** Hayley A. McNamara, Mireille H. Lahoud, Yeping Cai, Jessica Durrant-Whyte, James H. O’Connor, Irina Caminschi, Ian A. Cockburn

**Affiliations:** ^1^ Department of Immunology and Infectious Disease, The John Curtin School of Medical Research, The Australian National University, Canberra, ACT, Australia; ^2^ Division of Animal Physiology and Immunology, School of Life Sciences Weihenstephan, Technical University of Munich, Freising, Germany; ^3^ Department of Biochemistry and Molecular Biology, Monash Biomedicine Discovery Institute, Monash University, Clayton, VIC, Australia

**Keywords:** B cell, dendritic cells, *Plasmodium*, T cell help, circumsporozoite (CS) protein, malaria, plasmablasts, cell fate acquisition

## Abstract

Upon encountering cognate antigen, B cells can differentiate into short-lived plasmablasts, early memory B cells or germinal center B cells. The factors that determine this fate decision are unclear. Past studies have addressed the role of B cell receptor affinity in this process, but the interplay with other cellular compartments for fate determination is less well understood. Moreover, B cell fate decisions have primarily been studied using model antigens rather than complex pathogen systems, which potentially ignore multifaceted interactions from other cells subsets during infection. Here we address this question using a *Plasmodium* infection model, examining the response of B cells specific for the immunodominant circumsporozoite protein (CSP). We show that B cell fate is determined in part by the organ environment in which priming occurs, with the majority of the CSP-specific B cell response being derived from splenic plasmablasts. This plasmablast response could occur independent of T cell help, though gamma-delta T cells were required to help with the early isotype switching from IgM to IgG. Interestingly, selective ablation of CD11c^+^ dendritic cells and macrophages significantly reduced the splenic plasmablast response in a manner independent of the presence of CD4 T cell help. Conversely, immunization approaches that targeted CSP-antigen to dendritic cells enhanced the magnitude of the plasmablast response. Altogether, these data indicate that the early CSP-specific response is predominately primed within the spleen and the plasmablast fate of CSP-specific B cells is driven by macrophages and CD11c^+^ dendritic cells.

## Introduction

Successful vaccines rely upon the induction of robust B cell responses (1). These responses vary according to the cell fate that a B cell adopts following activation. A B cell may differentiate into an antibody secreting cell (ASC) that is short-lived (plasmablast– PB), a long-lasting ASC (plasma cell), a memory B cell, or a become a germinal centre (GC) B cell. A GC B cell can undergo rounds of affinity maturation before exiting the GC as a memory cell or plasma cell. Enforcement of these fates are driven by key transcription factors, such as Blimp1 for ASCs (2–4), Bcl-6 for GC B cells (5) and potentially Bach2 for memory B cells (6). However, the exact environmental factors which initially direct the fate of a newly activated B cells remain somewhat unclear. The influence of the B cell receptor (BCR) affinity in this process has been focused upon by many studies and seems to indicate that higher affinity BCRs form larger plasmablast populations (7–9), whilst lower affinity BCRs populate the memory B cell pool (9, 10). However, aside from BCR affinity, the extent to which interactions with other cellular compartments, including antigen presenting cells (APCs) and different T cell subsets, shape B cell fate has been less intensively examined.

One difficulty in determining the role of other cellular subsets in shaping the B cell response is the variety of cells there are involved. B cell priming often occurs within the spleen or lymph nodes and these alternative sites of priming host different populations of antigen presenting cells (APCs) (11, 12) and, in the case of the spleen, unique B cell populations such as marginal zone cells (MZ) (13). Indeed, within the spleen alone distinct populations of dendritic cells (DCs) have been observed to provide selective help for T cell responses (14–17). DCs also interact with B cells, having been found to be important in the initial stages of T-dependent responses (18–21). Thus, it seems possible that differences with APCs and the microenvironment in which B cells are primed can mediate crucial differences in the fate of these primed B cells, potentially affecting their capacity to provide long-lasting humoral protection.

While some studies have been conducted on the processing and presentation of antigens to B cells, much of this work has been done using proteins or immune complexes as immunogens (22–24). To understand how pathogen antigen is presented to B cells and how it affects cell fate decision we used a *Plasmodium* (malaria) immunization model. Malaria is caused by parasites of the genus *Plasmodium*, however protection against malaria can be conferred by immunization with irradiated sporozoites (25, 26). Irradiated sporozoites cannot divide but remain metabolically active and prime antibody and T cell responses which block infection in the liver, the first target of infection (27, 28). To assist with tracking of the *Plasmodium*-specific B cell response we utilised the established Igh^g2A10^ mouse model, an Ig-knockin mouse line which has elevated numbers of B cells specific to the immunodominant *Plasmodium falciparum* antigen *Pf*CSP (29). Use of these trackable cells allowed us to monitor the immune response in host mice lacking select immune compartments, and in a reductionist approach to dissect the crucial components of the early *Pf*CSP-specific B cell response.

## Results

### The Plasmablast Response Predominately Occurs in the Spleen Rather Than Lymph Nodes

**Figure 1:**
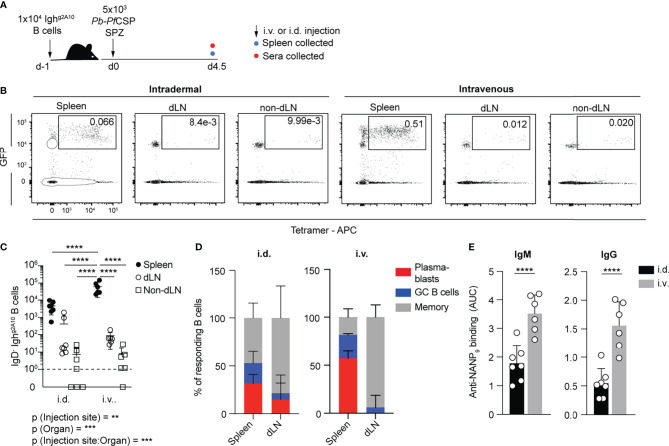
Splenic plasmablasts dominate the early response, regardless of immunization route. C57BL/6 mice received 1x10^4^ Igh^g2A10^ cells prior to intradermal (i.d.) or intravenous (i.v.) immunization with 5x10^3^ *Pb-Pf*CSP SPZ, 4.5 days later spleen and blood were taken for analysis. **(A)** Schematic of experiment. **(B)** Representative plots showing the proportion of total Igh^g2A10^ B cells recovered in spleen, draining lymph nodes (dLN) and non-draining lymph nodes (non-dLN) on day 4.5 post-immunization. Total Igh^g2A10^ B cells pre-gated on singlets, lymphocytes, Dump-negative and B cells [CD19^+^ ± CD138^+^]. **(C)** Quantification of the IgD- Igh^g2A10^ B cells recovered from tissues day 4.5 post-immunization. **(D)** Day 4.5 B cell subsets as a proportion of total IgD- Igh^g2A10^ cells. **(E)** Serum titres of anti-*Pf*CSP antibodies quantified by ELISA and presented as area under the curve (AUC). Data are pooled from two similar experiments, with 3-4 mice per experimental group. Analyses were performed using linear mixed models, with injection site and organ set as fixed experiment effects and the different experiments blocked to account for random effects. For **(C)** the overall p-value for each fixed effect and their interaction is shown under the graph and the pairwise values derived from post tests are indicated on the graph itself. Abbreviations for p values are: p < 0.01 = **; p < 0.001 = ***; p < 0.0001 = ****.

Our initial studies focused on how the route of immunization affects the outcome of the B cell response to *Plasmodium*. Vaccination with the current whole-parasite vaccine candidate, PfSPZ uses intravenous (i.v) inoculation to induce strong immune responses (26, 30) with T cell responses primarily in the spleen (31). However, it has previously been shown that during natural transmission the majority of sporozoites are deposited in the skin (32) and T cell responses to natural infection are primed in the skin draining lymph nodes as well as the spleen (27). Accordingly, we compared the response of *Pf*CSP-specific B cells within the spleen and skin-draining lymph nodes to i.v or intradermal (i.d) immunization. As the number of *Pf*CSP-specific B cells in naïve animals is low, we utilized the Igh^g2A10^ knock-in mouse model, which has elevated numbers of *Pf*CSP-specific B cells that facilitate easy tracking of B cell responses (29). 1x10^4^ Igh^g2A10^ B cells were adoptively transferred into congenic recipients one day prior to immunization with gamma-radiation attenuated *P. berghei* sporozoites that had been engineered to express *Plasmodium falciparum* CSP (33). Animals were immunized i.v *via* the trail vein or i.d in the ear pinna. At day 4.5 post-immunization the B cell response in the draining lymph nodes (dLNs; auricular and cervical), non-draining lymph nodes (non-dLN; inguinal) and spleen was examined by flow cytometry (**Figure S1** and **Figure 1A**). Adoptively transferred cells could be identified by ubiquitous GFP+ expression. Immunization *via* the i.d route resulted in markedly lower overall Igh^g2A10^ B cell responses than i.v immunization (**Figures 1B, C**), though both immunization methods found the largest response within the splenic B cell compartment (**Figures 1B, C**). This suggests that a significant proportion of the sporozoites inoculated within the ear had entered the vasculature and travelled to the spleen. Strikingly the response in the spleen after i.v immunization was heavily skewed towards the PB response at day 4.5. Even after i.d immunization the PB response was notably much more prominent in the spleen than the dLN, and serum levels of anti-*Pf*CSP antibodies correlated with PB numbers (**Figures 1D, E**). Collectively these data suggest that B cell responses, and in particular short lived PB responses, are preferentially primed in the spleen even after i.d. immunization.

### The Early Plasmablast Response Is Mainly Derived From Follicular B Cells

**Figure 2:**
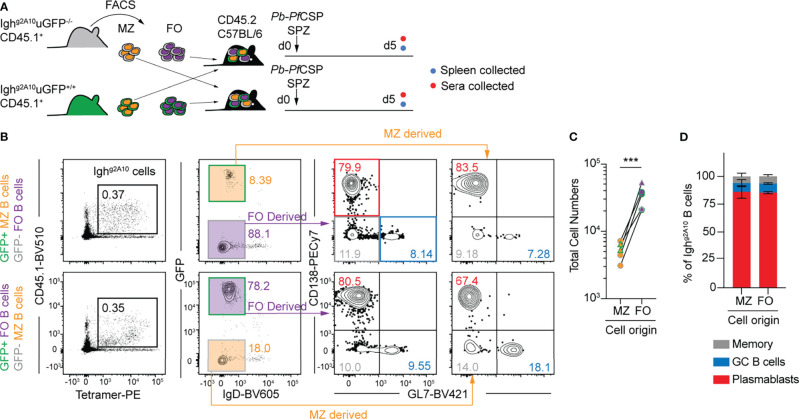
Marginal zone and follicular B cells contribute to the early *Pf*CSP-specific B cell response. **(A)** Schematic depicting the experiment strategy for co-transfer of 5 x 10^3^ marginal zone (MZ) and follicular (FO) Igh^g2A10^ B cells prior to immunization with 5 x 10^4^ *Pb-Pf*CSP SPZ with splenic immune response assessed at day 5 post-immunization. **(B)** Representative flow cytometry gating plots for identification of Igh^g2A10^ B cells 5 days post-immunization, pre-gated upon singlets, lymphocytes, Dump-negative, B cells [CD19^+^ ± CD138^+^]. Cells deriving from MZ or FO can be identified by expression of GFP. The plasmablast (PB) or germinal centre (GC) phenotype of these cells is depicted by subsequent staining for CD138 or GL7. **(C)** Quantification of the total number of responding Igh^g2A10^ B cells 5 days post-immunization, highlighting the original subset they were derived from. **(D)** B cell phenotype as a percentage of the total responding Igh^g2A10^ B cells. Results from two similar transfer experiments with at least 2 mice per group. Analyses were performed using a linear mixed model, cell type set as a fixed experiment effect and the different experiments blocked to account for random effects. Abbreviations for p values are: p < 0.001 = ***.

The murine spleen contains a unique population of marginal zone (MZ) B cells which reside at the border of the red pulp and white pulp and so are strategically positioned to respond to blood-borne pathogens. Notably, they respond rapidly to T-independent (TI) antigens (34) and we have previously shown that CSP is capable of inducing a TI B cell response (35). As MZ B cells are only localized within the spleen, we hypothesised that they were the driver of the *Pf*CSP-specific splenic PB response and would outcompete follicular (FO) B cells during the early response to immunization with *Pb*-*Pf*CSP SPZ. To test this hypothesis, congenically distinct MZ B cells and FO B cells were sorted from GFP+ and GFP- Igh^g2A10^ donor mice (**Figure S2A**), co-transferred into recipient mice and immunised with *Pb-Pf*CSP SPZ. As previously observed (29), MZ B cells in Igh^g2A10^ mice are elevated, comprising ~30% of the mature splenic B cell pool (**Figure S2A**). To ensure no bias from the GFP-status of the donor subset, co-transfers were carried out both with GFP+ and GFP- donors; donor cell populations were ~95% pure post sort (**Figure 2A**). To confirm the efficacy and stability of the transferred populations some recipient mice were left unimmunized, and the engraftment of the cells measured 2 days after transfer (**Figure S2B**). This revealed that follicular B cells engrafted ~3 fold better than MZ B cells (**Figure S2C**) and that there was some plasticity between the cell populations (**Figure S2D**) as has been reported previously (36). Five days post-immunization with *Pb*-*Pf*CSP SPZ, spleens were harvested and the expansion of each donor subset compared. In opposition to our hypothesis that MZ would dominate the PB response, we found that FO-derived B cells expanded to a greater extent than those from the MZ (**Figures 2B, C**). Allowing for differences in engraftment (**Figure S2C**) we estimate that FO B cells expanded ~500 fold while MZ B cells expanded ~300 fold after immunization. Furthermore, there was no bias in the differentiation of the responding B cells in each donor subset, with the overwhelming majority becoming PBs regardless of the original B cell type, or the GFP status of the donor animal (**Figure 2D**). Altogether, these data show that the early PB response to sporozoite antigen is not driven by MZ B cells in the spleen. The finding that there is some plasticity in the transferred populations (**Figure S2D**) is unlikely to affect this conclusion: if FO B cells became MZ B cells upon transfer and these were the source of the PB response by transferred FO cells we would expect the overall response by FO B cells to be smaller, but the opposite is true. Given the temporal distinction between these splenic subsets, this raises the question as to the manner in which these CSP-specific FO B cells encounter antigen from *Pb-Pf*CSP SPZ and whether T cell help is involved in shaping this response.

### Gamma-Delta T Cells Support Isotype Switching of CSP-Specific Plasmablasts

**Figure 3:**
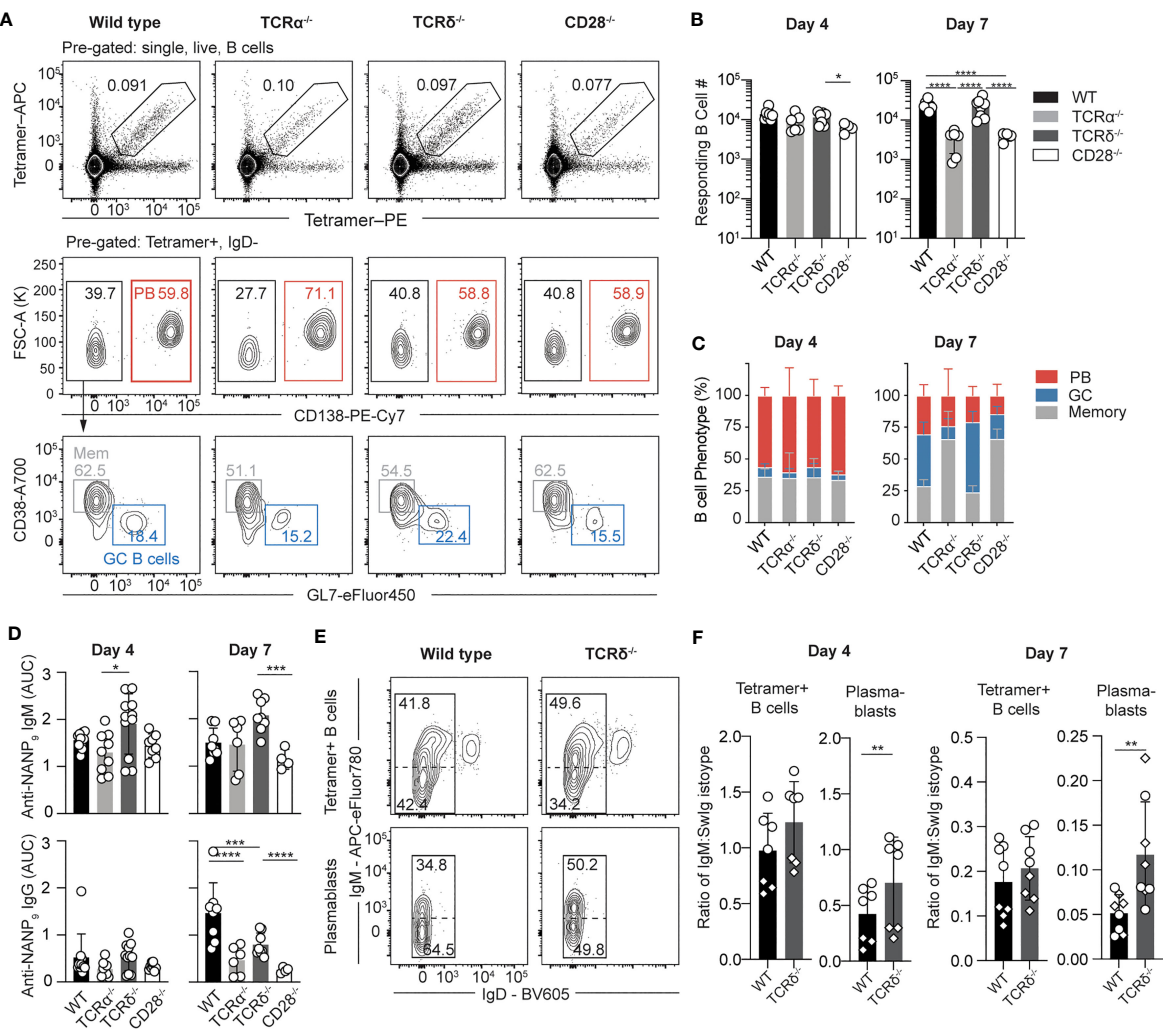
The early *Pf*CSP-specific B cell response is largely T-independent. TCRα^-/-^, TCRδ^-/-^, CD28^-/-^ and wild type (C57BL/6 –WT) mice were immunized with 5 x 10^4^ *Pb-Pf*CSP SPZ i.v. and the B cell and antibody response assessed 4 and 7 days post-immunization. **(A)** Representative flow cytometry plots showing the gating strategy used for identification of Igh^g2A10^ B cells and subsequent B cell populations. Pre-gated upon singlets, lymphocytes, Dump-negative, B cells [CD19^+^ ± CD138^+^]. **(B)** Quantification of total splenic Igh^g2A10^ B cell numbers from flow cytometry data at day 4 and 7 post-immunization. **(C)** Phenotype of Igh^g2A10^ B cells. **(D)** IgM and IgG anti-*Pf*CSP levels detected from sera *via* ELISA, depicted as area under the curve (AUC). **(E)** Ig isotype profile of Igh^g2A10^ B cells at day 4 post-immunization. Profiles depict both total Tetramer+ B cells or the plasmablast (PB) subset in WT or TCRδ^-/-^ host mice. **(F)** Ratio of IgM to SwIg B cells at day 4 and 7 after immunization, as gated according to **(E)** Data pooled from two independent experiments with 3-4 mice per group. Data for D includes an additional independent experiment with 3-4 mice per group. Mean ± s.d shown. Analyses were performed using a linear mixed model, with day and genotype set as fixed experiment effects and the different experiments blocked to account for random effects between experiments. Abbreviations for p values are: ns, not significant; p < 0.05 = *; p < 0.01 = **; p < 0.001 = ***; p < 0.0001 = ****.

In addition to MZ B cells, gamma-delta T cells are also overrepresented in the spleen. Gamma-delta T cells are an innate-like populations of T cells that carry a restricted repertoire of gamma-delta T cell receptors (TCRs). Notably, these cells have been shown to respond robustly to *Plasmodium* sporozoites, providing support for CD8 T cells responses (30, 37). Using CD28^-/-^ mice, we have previously shown that the response to sporozoites was largely T-dependent, albeit with a small early T-independent (TI) PB response (35). To clarify the contributions of gamma-delta and conventional alpha-beta T cell help to the early *Pf*CSP-specific B cells response, TCRα^-/-^, TCRδ^-/-^, and CD28^-/-^ mice were immunized with *Pb-Pf*CSP SPZ and the B cell response was tracked using antigen specific tetramer probes across the following days by flow cytometry (**Figures 3A–C**). At day four a strong PB response was observed in all groups of mice (**Figure 3C**), consistent with our previous observations of an early T-independent PB response to CSP. However, by day seven post-immunization the total B cell response in the TCRα^-/-^ and CD28^-/-^ mice was greatly reduced compared with the wild type (C57BL/6) and TCRδ^-/-^ (**Figure 3B**), indicating that at this time conventional CD4 T cell help was facilitating the expansion of *Pf*CSP-specific B cells. Responses in TCRδ^-/-^ mice –which retain the conventional CD4 helper ability – largely resembled the response of wild type mice, with robust GCs forming by day seven. However, when the sera of the mice was analysed to quantify the amount of anti-*Pf*CSP antibodies present, the TCRδ^-/-^ mice exhibited elevated titres of IgM (**Figure 3D**), which were higher than the wild type (though this did not reach statistical significance). This elevated IgM was still detectable in the serum at day seven (**Figure 3D**); furthermore, the TCRδ^-/-^ mice exhibited reduced titres of IgG compared to the wild type controls.

As the number of PBs were not significantly different within the wild type and TCRδ^-/-^ groups, the isotype of the responding cells was compared within the total tetramer+ B cell pool and the PB subset (**Figures 3E, F**). The TCRδ^-/-^ mice exhibited an increased percentage of IgM+ B cells in the PB population compared to WT mice, evidenced by the increased ratio of IgM to switched Ig (SwIg) cells. This data suggests that gamma-delta T cells play a role in helping B cells switch isotype during the early PB response.

### The PfCSP-Specific Plasmablast Response Is Primed Independent of MHC-II

**Figure 4:**
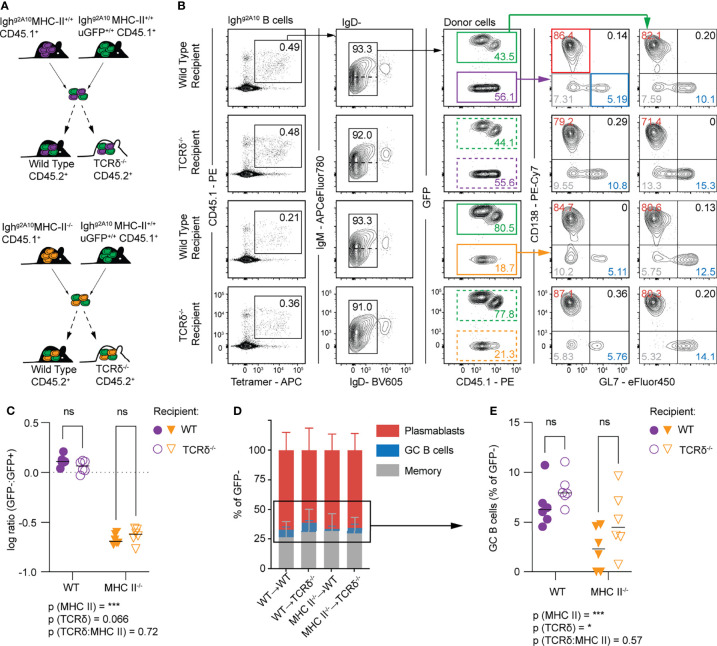
Conventional T cells provide help for GC B cell responses to sporozoites. Wild type (C57BL/6–WT) or TCRδ^-/-^ mice received 1 x 10^4^ Igh^g2A10^ cells which were, depending on group, a combination of wild type and MHC-II^-/-^ cells, prior to immunization with 5x10^4^ *Pb-Pf*CSP SPZ. Four days post-immunization the B cell response was analysed by flow cytometry. **(A)** Schematic depicting experimental strategy for co-transfer of MHC-II^+/+^ (WT) Igh^g2A10^ B cells and MHC-II^-/-^ Igh^g2A10^ B cells. **(B)** Representative flow cytometry gates for identification of Igh^g2A10^ B cells 4 days post-immunization, pre-gated upon singlets, lymphocytes, Dump-negative and B cells [CD19^+^ ± CD138^+^]. Co-transferred B cells can be distinguished by expression of GFP. Phenotype of these B cells subsequently identified by GL7 (GC) or CD138 (PB) expression. **(C)** Ratio between the total number of expanded Igh^g2A10^ B cells from within the co-transferred population, compared for expansion within WT or TCRδ^-/-^ host mice. **(D)** Phenotype of Igh^g2A10^ B cells as a percentage of total GFP- cells. **(E)** GC Igh^g2A10^ B cells from within co-transferred population as a percentage of total GFP- B cells compared for expansion within WT or TCRδ^-/-^ host mice. Data pooled from two independent experiments, with 3 mice per group. Analyses were performed using a linear mixed model, with recipient and donor genotypes set as fixed experiment effects and the different experiments blocked to account for random effects. For **(C, E)** the overall p-value for the interaction of the fixed effects is shown under the graph and the pairwise values derived from post tests are indicated on the graph itself. Abbreviations for p values are: ns, not significant; p < 0.05 = *; p < 0.001 = ***.

Because robust PB responses are seen after sporozoite immunization of both TCRα^-/-^and TCRδ^-/-^ mice, we wanted to know if there was some redundancy between these two T cell subsets. To address this question we utilised Igh^g2A10^ B cells which lacked MHC-II (MHC-II^-/-^) which were co-transferred with GFP+ MHC-II intact Igh^g2A10^ B cells (WT) into congenically distinct TCRδ^-/-^ and wild type recipients (**Figure 4A**). Control transfers of cells from MHC-II sufficient littermates were performed to verify that any differences were attributable to MHC-II expression rather than other variances in the background of donor mice e.g. expression of GFP (**Figure 4A**). Animals were immunised with *Pb-Pf*CSP SPZ and analysed by flow cytometry four days later (**Figure 4B**). In agreement with our findings in TCRα^-/-^ mice, MHC II^-/-^ Igh^g2A10^ B cells expanded after immunization, however this expansion was significantly less than the expansion of intact control cells (**Figure 4C**). Importantly, the presence of gamma-delta T cells in the recipient mice had no significant effect on the magnitude of the response by either MHC II^-/-^ Igh^g2A10^ or WT Igh ^g2A10^ B cells (**Figure 4C**).

We further examined the cell fate of the WT and MHC II^-/-^ Igh^g2A10^ cells in WT and TCRδ^-/-^ mice. Notably, the MHC II^-/-^ Igh^g2A10^ cells were less likely to differentiate into GL7^+^ GC precursor cells (**Figures 4D, E**), consistent with a requirement for T cells in initiating the GC reaction. Perhaps more surprisingly, cells were slightly more likely to become GC B cells in TCRδ^-/-^ mice, consistent with these cells supporting the early PB response (**Figures 4D, E**).

### A Type-2 T-Independent Response Drives the Early CSP-Specific Plasmablast Population

TI B cell responses can be separated into two types: Type 1 (TI-1), in which the co-stimulatory signal is replaced by the engagement of Toll-Like receptors (TLRs) and other Pattern-associated molecular pattern (PAMP) sensors, and Type 2 (TI-2) when highly repetitive epitopes are bound by multiple BCRs which results in BCR crosslinking and induces strong downstream signaling that overrides the need for a costimulatory signal. Given the highly repetitive nature of the CSP, it was hypothesized that the mechanism of the *Pf*CSP-specific TI response was TI-2. This was further supported by the fact that the early plasmablast response was derived from FO B cells rather than MZ B cells, which generally drive TI-1 responses.

**Figure 5:**
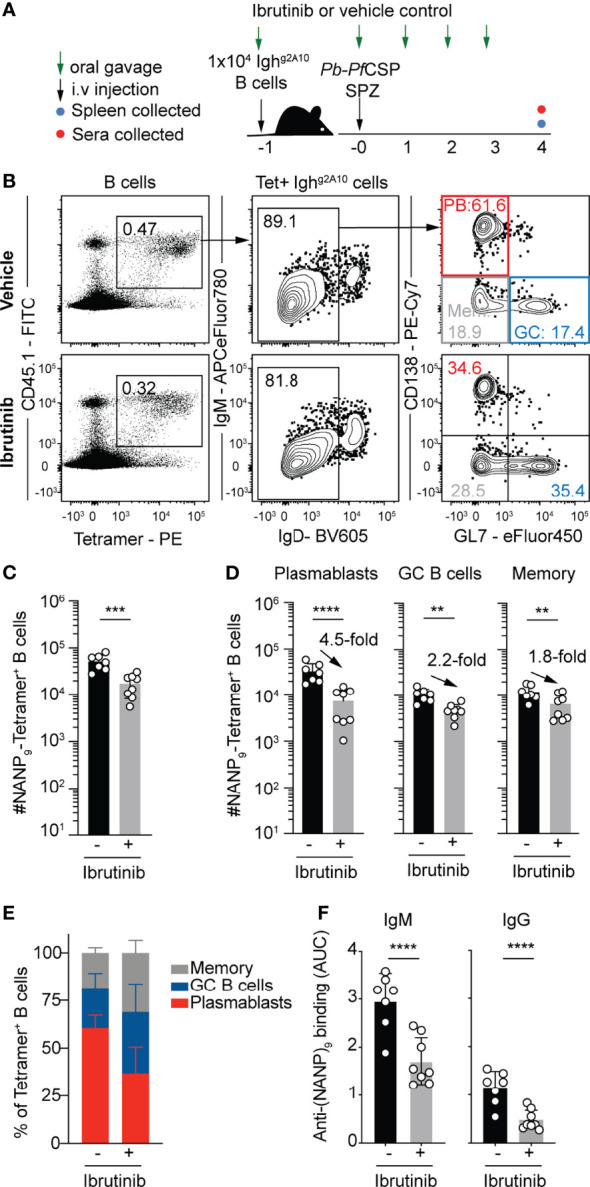
Pharmacologically suppressing TI-2 responses suppresses the *Pf*CSP-specific plasmablast response. 1x10^4^ Igh^g2A10^ cells were transferred to mice prior to immunization with 5x10^4^ *Pb-Pf*CSP SPZ in the presence or absence of Ibrutinib treatment, with splenic B cell and antibody responses assessed on day 4 post immunization. **(A)** Schematic of experimental strategy. **(B)** Representative flow cytometry plots for the gating of Igh^g2A10^ B cells at 4 days post-immunization. Plots are pre-gated upon singlets, Dump-negative and B cells [CD19^+^ ± CD138^+^]. **(C)** Quantification of total responding Igh^g2A10^ B cells. **(D)** Quantification of responding Igh^g2A10^ B cells of the plasmablast (PB), germinal centre (GC) and memory phenotype. **(E)** The proportion of each B cell phenotype as a percentage of the total Igh^g2A10^ population. **(F)** Serum titres of anti-*Pf*CSP antibodies at day 4 post-immunization, quantified by ELISA and presented as area under the curve (AUC). Data from two independent experiments, with 3-4 mice per group. Statistical analysis performed by linear mixed model, with different experiments accounted for as a blocking factor for random effects. Abbreviations for p values are: p < 0.01 = **; p < 0.001 = ***; p < 0.0001 = ****.

To directly assess the TI mechanism involved, the pharmaceutical agent Ibrutinib was utilized. Ibrutinib is a potent inhibitor of Bruton’s tyrosine kinase (BTK), a key protein in the downstream signaling of the BCR. Inhibition of BTK dampens the signaling of BCRs, and functional BTK is crucial for TI-2 responses to occur (38). In practice, this will largely inhibit the response of B cells activating by BCR cross-linking, but should allow B cells which engage T cell co-stimulation to still respond. Accordingly, naïve wild type mice were immunized with *Pb-Pf*CSP SPZ whilst receiving daily treatment with Ibrutinib (12.5mg/kg) or a vehicle control (**Figure 5A**). After four days, flow cytometry analysis of splenocytes revealed animals which were treated with Ibrutinib had significantly reduced numbers of *Pf*CSP-specific B cells (**Figures 5B, C**). Strikingly, the reduction in B cell numbers was primarily driven by a reduction in the PB response which contracted around 5-fold in the Ibrutinib treated group, while GC and memory cells only declined by around 2-fold (**Figure 5D**), leading them to comprise a greater proportion of responding B cells in drug treated mice (**Figure 5E**). This reduction in the PB response was reflected in the reduced titres of IgM and, to a lesser extent IgG, in the sera (**Figure 5F**). Coupled with the previous studies supporting with likelihood of a TI-2 response (35), these data suggest that the bulk of the early *Pf*CSP response is a TI-2 response. The data further support the hypothesis that strong BCR signaling favors a PB response.

### CD11c+ Cells Have an Influential Role in Directing the Plasmablast Fate of Early Responding B Cells

**Figure 6:**
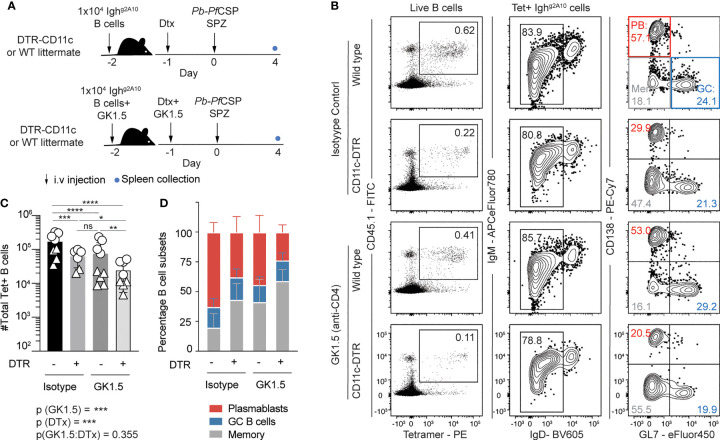
Depletion of CD11c+ dendritic cells impairs plasmablast differentiation after sporozoite immunization. Mice received treatment with Diptheria toxin (Dtx) prior to transfer of 1x10^4^ Igh^g2A10^ B cells and immunization with 5x10^4^ *Pb-Pf*CSP SPZ. Selected mice were also depleted of CD4+ T cells with GK1.5 treatment at days -2 and -1, immune responses were assessed 4 days post-immunization. **(A)** Schematic of experimental protocol. **(B)** Representative flow cytometry plots showing the gating of Igh^g2A10^ B cells in each condition. Plots are pre-gated upon singlets, Dump-negative and B cells [CD19^+^ ± CD138^+^]. **(C)** Quantification of *Pf*CSP-specific B cells from spleens of mice four days after immunization and phenotype **(D)** of Igh^g2A10^ B cells as a percentage of total Igh^g2A10^ cell number. Means ± s.d shown. Data pooled from two independent experiments, with 4-5 mice per group. Different icon shapes distinguish different experiments. Analysis performed by a linear mixed model, with different experiments set as a blocking factor to account for random effects between experiments. For **(C)** the overall interaction between fixed effects is noted with the p-value underneath the main figure, while the pairwise values derived from post tests are indicated on the graph itself. Abbreviations for p values are: ns, not significant; p < 0.05 = *; p < 0.01 = **; p < 0.001 = ***; p < 0.0001 = ****.

Dendritic cells (DCs) are capable of presenting intact antigen which can be recognized by B cells (19), have been observed to directly activate B cells (39) and initiate an antibody response (18). To investigate if DCs have a role in priming the *Pf*CSP-specific B cell response, we immunized CD11c-DTR mice (**Figure 6A**), which express the simian diphtheria toxin receptor (DTR) under the CD11c promoter allowing for transient depletion of most conventional DCs upon administration of Diphtheria toxin (Dtx) (40). However, in addition to any direct B cell priming role, DCs may also support B cell responses by priming CD4 Tfh cells. To overcome this, we took advantage of the strong TI response to sporozoites and in select mice we not only depleted DCs but used the CD4 depleting GK1.5 antibody to remove CD4 T cell help. Finally, as a small percentage of B cells do express CD11c, to avoid any bias from their depletion in the endogenous B cell response in CD11c-DTR animals, Igh^g2A10^ B cells were adoptively transferred into Dtx treated animals prior to *Pb*-*Pf*CSP SPZ immunization.

Four days after immunization, flow cytometry analysis revealed there was a significant reduction in the number of antigen specific Igh^g2A10^ B cells in the spleens of DC depleted animals in comparison with wild type littermates (**Figures 6B, C**). Strikingly this effect was seen even in the absence of CD4 T cell help, indicating that splenic DCs can directly prime B cells (**Figures 6B, C**). When we examined the phenotype of the responding cells we found that the PB response declined around 4-fold after DC depletion in CD4 T cell-sufficient mice and 7-fold in CD4 T cell-deficient animals (**Figure S3A** and **Figure 6C**). In contrast, upon DC depletion the GC response declined only around 3-fold in both CD4 T cell sufficient mice and CD4 T cell deficient mice, leading to an overall reduction in the proportion of cells differentiating into PBs in DC depleted animals (**Figure S3B** and **Figure 6D**). These data suggest that DCs directly prime B cells to become PBs independently of CD4 T cell help. However, it has been noted that marginal zone macrophages are also depleted in the CD11c-DTR mouse model (41), and so we cannot exclude a contribution from these cells in priming PB responses.

### Depletion of Macrophages Results Profoundly Reduces the Plasmablast Response to Sporozoites

**Figure 7:**
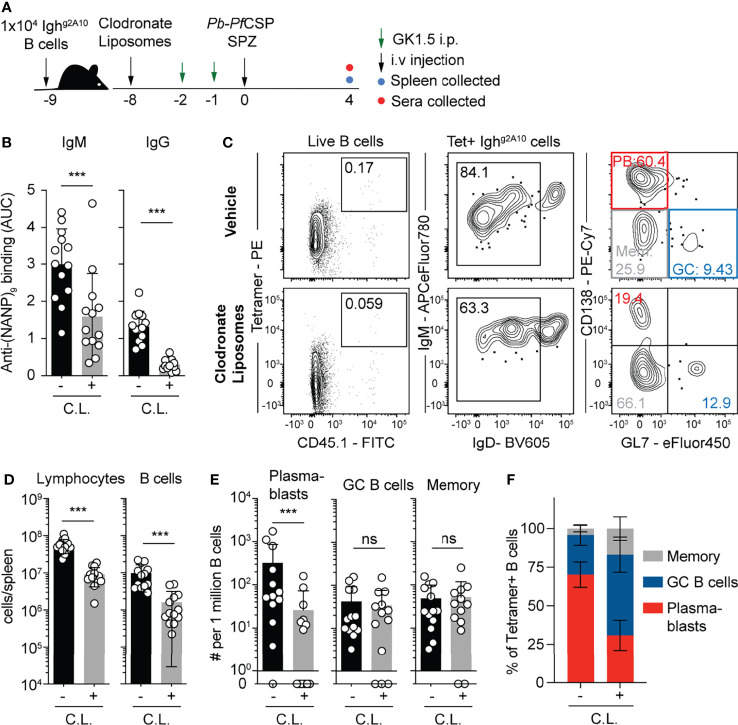
Depletion of macrophages suppresses the plasmablast response 1x10^4^ Igh^g2A10^ cells were transferred to mice prior to treatment with clodronate liposomes or vehicle alone 8 days prior to immunization with 5x10^4^ *Pb-Pf*CSP SPZ. All mice received two doses of CD4 depleting antibody (GK1.5) in the 2 days prior to immunization. Antibody and B cell responses were assessed on day 4 post immunization. **(A)** Schematic of experimental strategy. **(B)** Serum titres of anti-*Pf*CSP antibodies at day 4 post-immunization, quantified by ELISA and presented as area under the curve (AUC). **(C)** Representative flow cytometry plots for the gating of Igh^g2A10^ B cells at 4 days post-immunization. Plots are pre-gated upon singlets, Dump-negative and B cells [CD19^+^ ± CD138^+^]. **(D)** Quantification of total lymphocytes and B cells in the different treatment groups. **(E)** Quantification of responding Igh^g2A10^ B cells of the plasmablast (PB), germinal centre (GC) and memory phenotype. **(F)** The proportion of each B cell phenotype as a percentage of the total Igh^g2A10^ population. Data from three independent experiments, with 3-4 mice per group. Statistical analysis performed by linear mixed model, with different experiments accounted for as a blocking factor for random effects. Abbreviations for p values are: ns, not significant; p < 0.001 = ***.

As the CD11c-DTR system may also deplete CD11c-expressing macrophages in the spleen, we sought to separately assess the contribution of macrophages to the magnitude and quality of the B cell response to *Plasmodium* sporozoites. Accordingly we used clodronate liposomes (CL) to deplete macrophage populations in the spleen (42). We delivered CL or a vehicle control to mice 8 days prior to immunization with *Pb-Pf*SPZ, which we have previously shown causes the depletion of macrophage populations, but allows for the reconstitution of the dendritic cell compartment (43). To control for indirect effects on the B cell response mediated by CD4 T cells, all mice were depleted of CD4 T cells with GK1.5 (**Figure 7A**). Strikingly, both IgM and IgG responses to the repeat region of CSP were profoundly reduced in CL treated animals (**Figure 7B**). Flow cytometry analysis (**Figure 7C**) revealed profound reductions in the absolute numbers of total lymphocytes and B cells in the spleens of treated mice (**Figure 7D**). However, after normalizing for the number of B cells it was found that among transferred Igh^g2A10^ cells only the PB response was affected by CL depletion (**Figures 7E, F**), suggesting that the CL-depleted macrophages were critical for B cells to enter this particular B cell fate.

### Targeting of Antigen to Dendritic Cells Enhances the Plasmablast but Not the GC Response to the Circumsporozoite Protein

**Figure 8:**
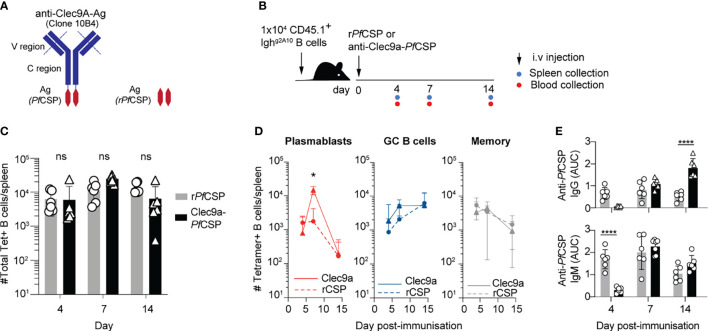
Targeting DCs for antigen presentation promotes the early plasmablast response. C57BL/6 mice received 1x10^4^ Igh^g2A10^ cells prior to immunization with r*Pf*CSP (2 μg) or anti-Clec9A-*Pf*CSP (10 μg) and assessment of B cell and antibody response at days 4,7 and 14 post-immunization. **(A)** Schematic of Clec9A conjugated to *Pf*CSP. **(B)** Schematic of experimental protocol. **(C)** Total number of responding Igh^g2A10^ B cells following immunization with anti-Clec9A-*Pf*CSP or recombinant *Pf*CSP (r*Pf*CSP). **(D)** Number of Igh^g2A10^ B cells of the plasmablast (PB), germinal centre (GC) and memory phenotype after immunization. **(E)** Serum levels of IgM and IgG anti-*Pf*CSP antibodies post-immunization, quantified by ELISA and presented as area under the curve (AUC). Data pooled from 2 independent experiments, with 3-4 mice per group. All statistical analyses performed by a linear mixed model incorporating day and immunization in the model as fixed effects, with individual experiments blocked to account for random variation. Abbreviations for p values are: ns, not significant; p < 0.05 = *; p < 0.0001 = ****.

While the previous experiment suggested that the lack of PB response observed in CD11c-DTR mice could also be attributed to the depletion of macrophages, we wished to further probe the possible role of DCs in priming *Pf*CSP-specific B cells. This was explored by attempting to enhance the priming of plasma cells with *Pf*CSP delivered directly to DCs by DC specific antibodies (44, 45). One of the most delivered approaches is the use of an antibody against the Clec9A lectin, expressed only on a subset of CD8+ DCs (46). Although the CD8+ DC subset is traditionally associated with cross-presentation and CD8 T cell responses, antigens-conjugated to anti-Clec9A (i.e. NP or OVA) have been observed to induce elevated antibody responses compared with non-targeted controls (47, 48). This enhancement can occur in the absence of any adjuvant. We therefore generated a novel anti-Clec9A-*Pf*CSP conjugate with recombinant *Pf*CSP (**Figure 8A**). To assess whether targeting *Pf*CSP directly to DCs enhanced the humoral response, C57BL/6 mice received Igh^g2A10^ B cells which were tracked after vaccination with anti-Clec9A-*Pf*CSP conjugate or a control of unconjugated r*Pf*CSP (**Figure 8B**). Whilst there was no significant difference in the total number of Igh^g2A10^ B cells responding, the group administered the anti-Clec9A conjugate had an elevated PB response at day seven (**Figures 8C, D**). This PB response is reflected in the ELISA titres, with enhanced IgM and IgG at days seven and fourteen (**Figure 8E**). Interestingly, r*Pf*CSP vaccinated mice appear to have an earlier IgM response at day four, significantly higher than the anti-Clec9A conjugate group. It is possible that the r*Pf*CSP may have been able to activate rapid TI responses by directly binding to BCRs as a soluble antigen, whilst that the anti-Clec9A conjugate required additional time to be processed by the DCs and presented to B cells. Coupled with the observation that the PB response is decreased in the absence of DCs (**Figure 6**), these data further suggest that DC-dependent priming directs B cells to the enter the PB fate.

## Discussion

In this study we have shown that the majority of the early *Pf*CSP-specific B cell response to *Pf*CSP SPZ immunization is derived from splenic PBs. This PB response was predominately T-independent, though gamma-delta T cells assist with early isotype switching from IgM to IgG. Interestingly, CD11c+ DCs were implicated in this PB response, as their selective ablation significantly reduced the splenic PB response, independent of the presence of helper CD4 T cells. Conversely, immunization approaches that directly targeted *Pf*CSP-antigen to DCs enhanced the magnitude of the PB response. Altogether, these data indicate that the early *Pf*CSP-specific response is predominately primed within the spleen and the plasmablast fate of *Pf*CSP-specific B cells is mediated directly by CD11c+ dendritic cells.

The factors involved in the choice a B cell makes between differentiation into a short-lived plasma cell, or a GC B cell with the capacity to confer long term protection, are poorly understood. Whilst earlier *in vitro* work suggested differentiation occurred in a stochastic manner (49), the recent narrative has largely focused upon how B cell affinity, and subsequent strength of BCR-mediated signalling, direct possible outcomes. Indeed, in murine systems it has been clearly demonstrated that in T-dependent B cell responses manipulating the BCR to a lower affinity (7, 8), or immunising with a lower affinity antigen (9), results in fewer antigen-responding B cells entering an ASC fate. Alternately, B cells with lower affinity for antigen will favour memory cell fates (9, 10). What has been largely neglected is an examination of how cellular interplay between B cells and other immune subsets can bias B cell fate differentiation. Strong conventional T cell help appears to favour differentiation into GC responses. In additional gamma-delta T cells drive the early isotype switching of PBs in response to sporozoite immunization. This interesting role of gamma-delta T cells is not unprecedented, as gamma-delta T cells have previously been observed to assist B cells with GC-independent isotype switching (50, 51). However, our results are the first demonstrating this occurrence in response to *Plasmodium* sporozoites, and help to explain why the early *Pf*CSP-specific PB response contains many isotype switched cells. Nonetheless, the relatively minor role that gamma-delta cells play in the B cell response to sporozoites is in striking contrast to the major role these cells appear to play in the development of protective CD8 T cell responses (37).

One of the most striking findings we observe is a direct role for CD11c+ DCs and macrophages in generating PBs specific for the malaria *Pf*CSP antigen. Notably, this occurs in a manner independent of CD4 T cell help, as depletion of CD4 T cells failed to negate the difference driven by the presence of DCs or macrophages. The DCs involved in determining this fate may be related to plasmablast-associated dendritic cells which were observed in an older study (52). These CD11c^high^ dendritic cells were found to be crucial for survival of extrafollicular PBs, and independent of T cell help. However, a later study has challenged this finding, concluding that maintenance and differentiation of plasma cells was independent of CD11c^high^ DCs (53). Their approach utilised elaborate bone marrow chimeras and DC depletion with the murine CD11c-DTR, as we have used in our study. We clearly see a difference mediated by the removal of CD11c+ DCs, though it should be noted that in the CD11c-DTR model a histological study found that in addition to clearance of CD11c+ DCs, Dtx treatment also resulted in clearance of MZ and metallophilic macrophages from the spleen (41). Importantly we verified the importance of DCs in mediating PB differentiation by directly targeting them for antigen presentation with the use of anti-Clec9A conjugated *Pf*CSP. Use of anti-Clec9A conjugated antigen recently revealed that conventional DCs interact extensively with B cells at the splenic T-B border (21). The role of DCs in the B cell response to pathogens has largely been considered linked to their role in priming helper T cells, which then influence B cells. Here, we identify that this DC-targeted immunization drives the expansion of *Pf*CSP-specific B cells towards a short-lived PB response. Further examination of how the response is exclusively affected by the removal of DCs could be refined with the use of the zDC-DTR mice, for which DTR expression is under *Zbtb46* and is restricted to the DC lineage (54).

Macrophages have previously been shown to play key roles in the initiation of responses to *Plasmodium* sporozoites. Notably subcapsular sinus (SCS) macrophages, which are the lymph node equivalent of marginal zone macrophages, trap sporozoite antigen before it is passed to DCs for presentation (55). SCS macrophages have previously been implicated in the initiation of B cell responses to particulate antigen (24, 56), though their role in determining B cell fate was not examined. Subsequent studies have however suggested that SCS may drive the extrafollicular proliferation of memory B cells and their differentiation into antibody secreting cells (57). Separately, SIGN-R1+ macrophage populations have been reported to drive GC B cell responses (58), though this effect was apparently mediated through efficient priming of the Tfh response, rather than a direct effect on B cell population.

It is important to study B cell fate choices in the context of complex pathogens as well as protein immunogens. In the context of malaria, it was recently shown that the PB response acts as nutrient sink inhibiting the GC response (59), leading to poor infection outcomes. After sporozoite immunization in mice we see a very strong extrafollicular PB response, which mirrors what we have observed previously in humans (29, 60). If this response also occurs at the expense of a robust GC reaction this may explain the relatively poor affinity maturation observed in response to *Plasmodium* sporozoites (29, 61). Our data suggests that DC targeting, which has been proposed as a strategy for driving protective T cell responses (62) may alter the balance of B cell responses. Thus, our findings have important implications not only for understanding B cell fate decisions but also the design of vaccines to induce balanced T and B cell responses.

## Methods

### Mice and Ethics

All animal procedures were approved by the Animal Experimentation Ethics Committee of the Australian National University (Protocol numbers: A2016/17 and A2019/36). Mice were bred and maintained under specific pathogen free conditions in individually ventilated cages at the Australian National University and were 5-8 weeks old at the commencement of experiments. Female mice were preferentially used within experiments, but when males were used they were controlled for appropriately. Igh^g2A10^ mice have the germline-reverted heavy-chain V(D)J exon (*Ighv903, Ighd1-3* and *Ighj4*) of the murine *Pf*CSP-neutralizing monoclonal antibody 2A10 inserted upstream of the IgM locus, and these mice have been previously described (29). Some Igh^g2A10^ mice were crossed to ubiquitous GFP-expressing mice and were able to express GFP to facilitate tracking of transferred cells. Similarly Igh^g2A10^ mice (both GFP+ and GFP-) were backcrossed to the B6.SJL-*Ptprc^a^ Pepc^b^*/BoyJ (CD45.1) congenic strain to facilitate tracking in C57BL/6 hosts. MHC-II^-/-^ mice (63) were a kind gift from Dr Anselm Enders (The Australian National University). TCRα^-/-^ mice (64) were a kind gift from Dr Steve Daley (Monash University), TCRδ^-/-^ (65) and CD11c-DTR (40) mice were a kind gift from Dr Lynette Beattie and Prof Willian Heath (University of Melbourne).

### Parasites and Immunizations

Mice were immunized with 5x10^4^ *Pb*-*Pf*CSP SPZ crossed to a GFP background to facilitate the identification of infected mosquitoes (33, 66). Sporozoites were dissected by hand from the salivary glands of infected *Anopheles stephensi* mosquitoes and were irradiated (200kRad) using a MultiRad 225 (Flaxitron) irradiator prior to injection. Unless mentioned, immunizations were performed intravenously *via* the trail vein plexus.

For intradermal immunizations, irradiated sporozoites were concentrated according to the protocol (27), after-which 5x10^4^ were injected into the dermis of the back of the ear using a 10µl microsyringe (Hamilton). Injections were performed under a dissection microscope upon mice anaesthetized with Ketamine/Xylazine (100 mg/kg and 10 mg/kg, respectively).

### Generation of Clec9A Targeting Constructs and Immunizations

Plasmids encoding recombinant anti-Clec9A antibody clone 10B4 heavy and light chains in pcDNA3.1+ were generated by gene synthesis of codon-optimised antibody sequences as previously described (GeneArt, Thermo Fisher Scientific) (67). Antibody (Ab) constructs carrying the *Pf*CSP construct were generated by fusing the Ab heavy chain to a cDNA encoding an alanine linker and the antigenic sequence **(**AAARGSSSNTRVLNELNYDNAGTNLYNELEMNYYGKQENWYSLKKNSRSLGENDDGNNNNGDNGREGKDEDKRDGNNEDNEKLRKPKHKKLKQPGDGNPDPNANPNVDPNANPNVDPNANPNVDPNANPNANPNANPNANPNANPNANPNANPNANPNANPNANPNANPNANPNANPNANPNANPNANPNANPNANPNANPNANPNANPNKNNQGNGQGHNMPNDPNRNVDENANANNAVKNNNNEEPSDKHIEQYLKKIQNSLSTEWSPCSVTCGNGIQVRIKPGSANKPKDELDYENDIEKKICKMEKCSSVFNVVNSS.**).** The recombinant Ab-Ag carrying 2 Ag per Ab molecule (1 copy per heavy chain) were expressed in Freestyle 293F cells (Thermo Fisher Scientific) using 293Fectin (Thermo Fisher Scientific) and purified from culture supernatants using protein G as previously described. Ab specificity was confirmed by binding to cells transiently transfected with full length mouse Clec9A, as previously described (67). For immunizations, 10μg anti-Clec9A-*Pf*CSP conjugate was resuspended in PBS and administered i.v. To validate the effect of anti-Clec9A targeting, control mice were immunized i.v with the equivalent molar ratio of unconjugated r*Pf*CSP (2μg) in PBS.

### Treatment of Mice With Ibrutinib

Ibrutinib (SelleckChem) – an inhibitor of Bruton’s Tyrosine Kinase (BTK) – was dissolved in DMSO and stored at -80°C prior to use. Due to the sensitivity upon thawing, vials were thawed immediately before animal treatments and emulsified in castor oil *via* vortexing. Animals due to be treated with Ibrutinib were weighed prior to the beginning of their treatment. The Ibrutinib emulsion was administered *via* oral gavage at a dosage of 12.5mg/kg, every 24 hours until the end of the experiment. Control mice received an equivalent dose of vehicle (DMSO in castor oil) *via* oral gavage, every 24 hours until the end of the experiment.

### Treatment of Mice With GK1.5 Antibody

To transiently deplete CD4 T cells from mice, the anti-CD4 antibody GK1.5 (BioXcell) was used. Animals received two doses of antibody at 100μg/mouse in PBS *via* i.p injections on the two days prior to an experimental immunization. Control groups of mice received an equivalent dose of the isotype (LTF2; BioXcell) control alongside the CD4 depleted group.

### Depletion of Dendritic Cell and Macrophage Subsets

For depletion of dendritic cells, CD11c-DTR mice were treated with 100ng Diphtheria toxin (Dtx) (Sigma) *via* an i.p injection, one day prior to experimental immunizations. Upon initial reconstitution of DTx, the DC-depleting efficiency was confirmed by flow cytometry of splenocytes from CD11c-DTR mice which had received DTx i.p one day prior, and the absence of CD11c+ DCs compared to a C57BL/6 mouse which had received Dtx. Macrophages were depleted following i.v injection of clodronate liposomes (Liposoma BV) 8 days prior to immunization as described previously (42). Depletion of macrophages was confirmed by flow cytometry as shown previously (43).

### Cell processing and Flow Cytometry

Lymphocytes were isolated from the spleen and lymph nodes of mice and were prepared into single cell suspensions for flow cytometric analysis and sorting. Lymphocytes were isolated by mashing tissues over 70µm mesh filters. Red blood cells were lysed from cell suspensions with ACK lysis buffer (Sigma) and cells were washed twice with FACs wash prior to FC-block (Biolegend) and antibody staining. Cells were quantified during flow cytometry by the addition of CountBright Absolute Counting beads (Invitrogen) to sample suspensions. For instances where counting beads were unable to be used, quantification was performed by counting with a hemocytometer and percentages from flow cytometry. (NANP)_9_ tetramers were prepared in house by mixing biotinylated (NANP)_9_ peptide with streptavidin conjugated PE or APC (Invitrogen) in a 4:1 molar ratio. Live cells were always distinguished with the use of 7-AAD (Biolegend), which was included in the “Dump-gate” channel (which included the markers CD11b and GR1). Flow-cytometric data was collected on a BD Fortessa or X20 flow cytometer (Becton Dickinson) and analyzed using FlowJo software (FlowJo). A BD FACs Aria I or II (Becton Dickinson) machine was used for FACS sorting of cells.

### ELISA for Detection of Anti-CSP Antibodies

Concentrations of CSP-specific Abs in the sera of mice after immunization were measured using solid phase ELISA. Briefly, Nunc Maxisorp Plates (Nunc-Nucleon) were coated overnight with 1ug/ml streptavidin followed by binding of biotinylated (NANP)_9_ peptide for 1 hour. After blocking with 1% BSA, serial dilutions of the Abs were incubated on the plates for 1 hour and after washing, incubated with HRP conjugated anti-mouse IgG or anti-mouse IgM Abs (KPL). Plates were developed for 15-20 minutes with ABTS 2-Component Peroxidase Substrate Kit (KPL) and read at 405nm using a Tecan Infinite 200Pro plate reader. Responses were analysed as the area under the absorbance curve (AUC).

### Statistical Analysis

Details of specific statistical tests and experimental design are given in the relevant figure legends. Most experiments had 3-5 mice per group and were performed either in duplicate or triplicate. In most instances analysis was performed in R (The R Foundation for Statistical Computing) on the pooled data from all replicate experiments. Where data was pooled from multiple experiments, each experiment was included as a blocking factor in the analysis. Where data are plotted on a log-scale, data were log-transformed prior to analysis. For certain experiments blocking factors did not need to be accounted for and analysis was performed in GraphPad Prism 9. No blinding or randomization was performed; however all readouts (ELISA and flow cytometry) are objective readouts that are not subject to experimental bias.

## Data Availability Statement

The original contributions presented in the study are included in the article/**Supplementary Material**. Further inquiries can be directed to the corresponding author.

## Ethics Statement

All animal procedures were approved by the Animal Experimentation Ethics Committee of the Australian National University (Protocol numbers: A2016/17; 2019/36). All research involving animals was conducted in accordance with the National Health and Medical Research Council’s Australian Code for the Care and Use of Animals for Scientific Purposes and the Australian Capital Territory Animal Welfare Act 1992.

## Author Contributions

HAM: Conceptualization, Methodology, Formal Analysis, Investigation, Writing – Original Draft, Writing Editing and Review, Visualization. MHL: Investigation, Resources. JD-W: Investigation. YC: Investigation, Resources. JHO’C: Investigation; IC: Investigation, Resources. IAC: Conceptualization, Methodology, Writing – Original Draft, Writing Editing and Review, Visualization, Supervision, Project Administration, Funding Acquisition. All authors read and approved the manuscript prior to submission.

## Funding

This study was funded by a project grant (GNT1158404) from the National Health and Medical Research Council (Australia) to IAC.

## Conflict of Interest

MHL and IC are listed as inventors on patents related to Clec9A antibodies. All other authors declare that the research was conducted in the absence of any commercial or financial relationships that could be construed as a potential conflict of interest.

## Publisher’s Note

All claims expressed in this article are solely those of the authors and do not necessarily represent those of their affiliated organizations, or those of the publisher, the editors and the reviewers. Any product that may be evaluated in this article, or claim that may be made by its manufacturer, is not guaranteed or endorsed by the publisher.
