# Supplementary material for: Splenic Dendritic Cells and Macrophages Drive B Cells to Adopt a Plasmablast Cell Fate
Source: Front Immunol. 2022 Apr 12;13:825207. doi: 10.3389/fimmu.2022.825207 (PMC9039241; doi:10.3389/fimmu.2022.825207)
Supplement: Supplementary file 1 [file DataSheet_1.pdf]

Supplementary Information for:

Splenic dendritic cells and macrophages drive B cells to adopt a plasmablast cell fate

Hayley A. McNamara, Mireille H. Lahoud, Yeping Cai, Jessica Durrant-Whyte, James H. O'Connor, Irina Caminschi and Ian A. Cockburn

Supplementary Figure 1

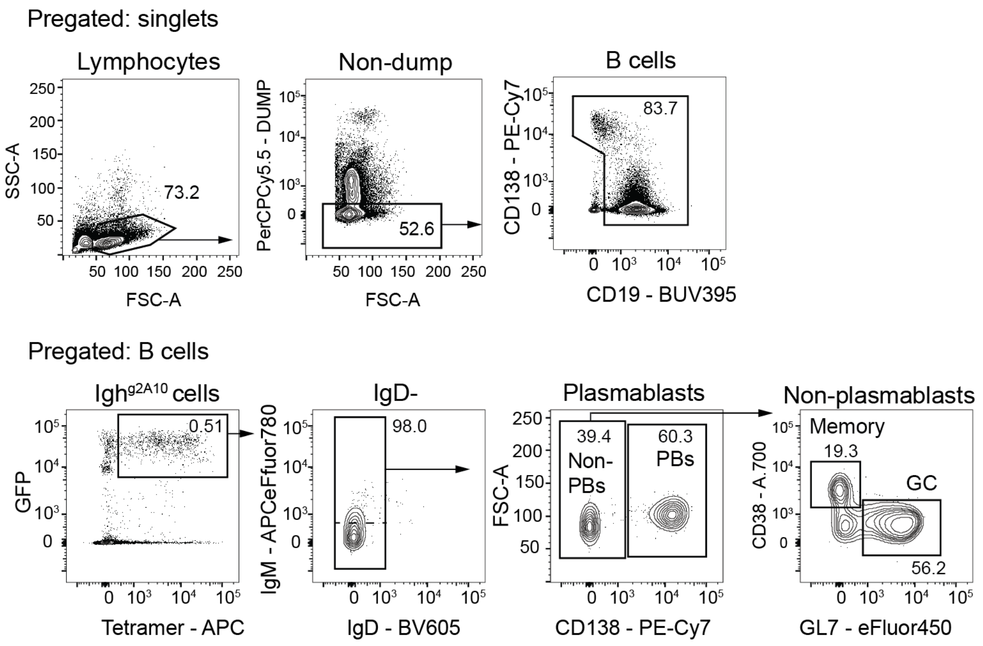

Figure S1. Gating strategy for *Pf*CSP-specific B cell subsets. The gating strategy used for the identification of *Pf*CSP-specific B cells and subsequent identification of activated B cells (IgD-), their division between plasmablasts (CD138+) and non-plasmablasts (CD138-), and the non-plasmablast populations of memory B cells (CD38+GL7-) and germinal centre B cells (CD38-GL7+). Identification of the transferred population of Igh^{g2A10} B cells was through congenic marker (CD45.1 Igh^{g2A10} cells transferred to a CD45.2 host mouse) or the GFP expression (Igh^{g2A10} cells from a mouse that expresses ubiquitous GFP).

Supplementary Figure 2

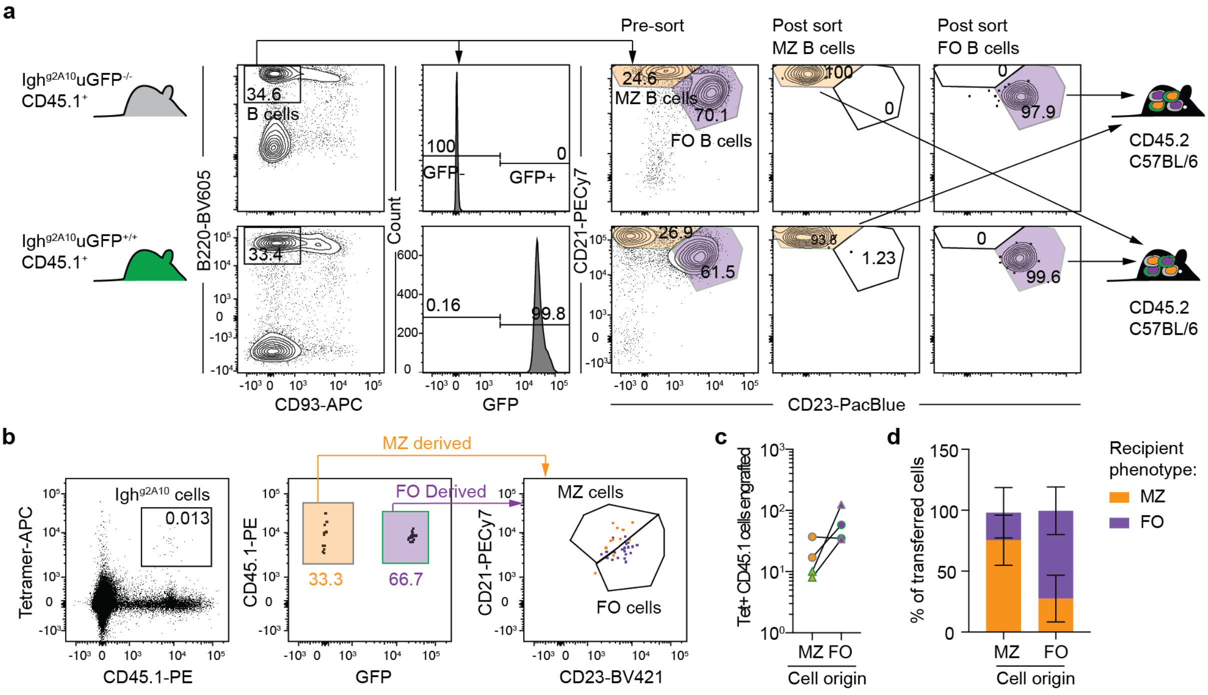

Figure S2. Marginal zone and follicular B cells retain their phenotype upon adoptive transfer. (A) Schematic of sort of marginal zone (MZ) and follicular (FO) Igh^{g2A10} B cells from GFP+ and GFP- donor mice. Gating of pre-sorted and post-sorted populations shown. (B) Representative flow cytometry plots 2 days post transfer of cells to recipient mice which were left unimmunised, to assess engraftment status. Prior to flow cytometry, magnetic enrichment of the donor cells was performed with CD45.1-PE staining and anti-PE magnetic beads, due to their small number in the naïve recipient spleen. (C) Number of cells engrafted in recipient mice from either MZ or FO donor cells 2 days post transfer. (D) Phenotypes of cells in recipient mice derived from either MZ or FO donor cells 2 days post transfer.

Supplementary Figure 3

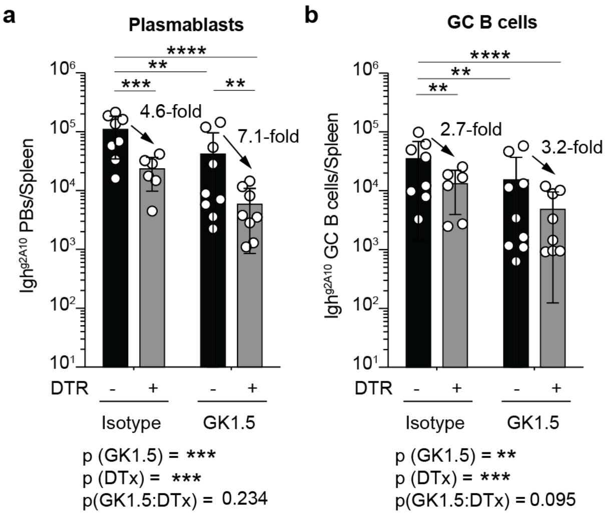

Figure S3. Depletion of CD11c+ dendritic cells impairs plasmablast more strongly than GC B cell differentiation after sporozoite immunization. Mice received treatment with Diptheria toxin (Dtx) prior to transfer of 1×10^4 Igh^{g2A10} B cells and immunization with 5×10^4 *Pb-Pf*CSP SPZ. Selected mice were also depleted of CD4 T cells with GK1.5 treatment at days -2 and -1, immune responses were assessed 4 days post-immunization. Quantification of (A) *Pf*CSP-specific Igh^{g2A10} plasmablasts or (B) *Pf*CSP-specific Igh^{g2A10} GC B cells from spleens of mice four days after immunization. Means ± s.d shown. Icons indicative of individual mice, with data pooled from two independent experiments. Analysis performed by a linear mixed model, with different experiments set as a blocking factor for random effects.
